# Control of Panama disease of banana by intercropping with Chinese chive (*Allium tuberosum* Rottler): cultivar differences

**DOI:** 10.1186/s12870-020-02640-9

**Published:** 2020-09-17

**Authors:** Zhenfang Li, Tong Wang, Chenling He, Kelin Cheng, Rensen Zeng, Yuanyuan Song

**Affiliations:** Key Laboratory of Ministry of Education for Genetics, Breeding and Multiple Utilization of Crops, College of Agriculture, Fujian Agriculture and Forestry University, Fuzhou, 350002 China

**Keywords:** Banana, Fusarium wilt disease, Chinese chive, Intercropping, 2-Methyl-2-pentenal, Cultivar

## Abstract

Panama disease (Fusarium wilt disease) caused by *Fusarium oxysporum* f. sp. *cubense* race 4 (FOC) severely threatens banana (Musa spp.) production worldwide. Intercropping of banana with *Allium* plants has shown a potential to reduce Panama disease. In this study, six cultivars of Chinese chive (*Allium tuberosum* Rottler) were selected to compare their differences in antifungal activity and active compounds. Three cultivars Duokang Fujiu 11, Fujiuhuang 2, and Duokang Sijiqing with higher levels of antifungal compounds were further used for intercropping with banana in the pots and field to compare their effects on growth and disease incidence of banana.

The six cultivars showed significant differences in antifungal activity against FOC mycelia growth in both leaf volatiles and aqueous leachates. The aqueous leachates displayed stronger antifungal activity than the volatiles. FJH cultivar showed the best inhibitory effect among all six cultivars. Contents of three main antifungal compounds dipropyl trisulfide (DPT), dimethyl trisulfide (DMT), and 2-methyl-2-pentenal (MP) in volatiles and aqueous leachates varied considerably among cultivars. Pot and field experiments showed that intercropping with three selected Chinese chive cultivars significantly improved banana vegetative growth, increased photosynthetic characteristics and yield but decreased disease incidence of Panama disease.

Our results indicate that intercropping with Chinese chive shows potential to reduce banana Panama disease and selection of appropriate cultivars is vital for effective disease control.

## Background

Banana (*Musa* spp.) is one of the world’s most important crops and it serves as a staple food in many developing tropical and subtropical countries [1]. It ranks next to rice, wheat, and maize in terms of its importance as a staple food crop [2]. Over 100 million metric tons of the crop are produced annually [3]. However, this crop production is severely hampered by Panama disease (also known as Fusarium wilt disease) caused by *Fusarium oxysporum* f. sp. *cubense* race 4 (FOC) [4], leading to extensive failure of commercial plantations in all banana-producing areas, including Asia, Central and South America, Africa, and Australia [5–7]. The soil-borne fungal pathogen colonizes and occludes the host xylem to cause vascular wilt [5, 6, 8]. So far not yet a practically effective approach of controlling this notorious disease in the field is available. Soil sterilization using fungicides has been used only for intensive agriculture in greenhouses on a limited scale [9, 10]. Environmentally friendly cultural practices and fungicides are urgently needed [11–13]. Fungicides are not able effectively to control this soil-borne pathogen in the field, for a variety of ecological reasons [14]. Nowadays, cultural practices have been tried with little success, for there has not been a suitably productive cultivar to selection or breeding for reducing the incidence of diseases caused by FOC [15]. Recently, crop rotation and microorganisms have shown promise for reducing the disease severity [16, 17].

Chinese farmers have practiced intercropping and rotation for disease control over 2000 years [18, 19]. Intercropping, which is defined as the growing of two or more crop species in proximity simultaneously during a growing season, is an agricultural practice that not only increases crop yield and stress resilience, but also reduces the incidence of microbial diseases. Chemicals produced by one plant in the intercropping system, can either directly inhibit microbial pathogens in the soils, or indirectly trigger resistance through the induction of the plant defense system in neighboring plants [20, 21]. The *Allium* plants are characterized with the production of many antimicrobial substances [21–24]. They are among the oldest medicinal herbs, used as antibacterial, antifungal, antioxidant and cytotoxic drugs [25]. In addition to many organosulphur compounds, mainly disulphides and trisulphides, *Allium* plants also produce non-volatile compounds such as spirostanol, furostanol and cholesterol saponin with antimicrobial, antifungal, antiplatelet aggregating, and cytotoxic activities [26–30]. Many *Allium* species such as garlic, onion and Chinese chive (*Allium tuberosum*) are widely cultivated for their spice and medicinal properties. Chinese chive is an important vegetable for making dumpling and kimchi in many Asian countries such as China and Korea. Chinese chive is now gaining increasing attention due to its diverse important pharmacological activities [28, 31]. Although many studies have been conducted for food and pharmacological purposes [24, 32], *Allium* plants as biological control agents for crop disease in the intercropping system and underlying mechanisms are scarcely addressed [33, 34]. The sequential cropping systems are becoming increasingly popular in all management practices for their environmental friendliness.

Recent studies indicated that banana intercropping with Chinese chive could effectively control Fusarium wilt of banana [31, 34, 35]. Our previous study showed that Chinese chive exhibited strong inhibitory effects on FOC through the release of organic volatiles, among which 2-methyl-2-pentenal, dimethyl trisulfide, dimethyl disulfide, dipropyl disulfide, and dipropyl trisulfide are the main active antifungal compounds [34]. However, crop varieties may markedly differ in their ability to produce antimicrobial chemicals [36].

In this paper, we selected six Chinese chive cultivars with obviously morphological difference and distinct characteristics to compare their antifungal activity against FOC and active compounds in the leaf volatiles and aqueous leachates of aerial parts. Three cultivars with higher contents of three antifungal compounds including dipropyl trisulfide (DPT), dimethyl trisulfide (DMT), 2-methyl-2-pentenal (MP) in the volatiles or aqueous leachates were further used in pot experiments to evaluate their potential in reducing disease incidence of Fusarium wilt disease of banana.

## Methods

### Chemicals

Three antifungal compounds including dipropyl trisulfide (DPT), dimethyl trisulfide (DMT), and 2-methyl-2-pentenal (MP) were obtained from Sigma-Aldrich (St. Louis, MO, USA). All solvents used were analytical or HPLC grade.

### Plant materials

The banana plant in this study was “Williams” (AAA genome, Cavendish subgroup) banana cultivar, which was purchased from Tianyang banana breeding Co. in Guangxi province in China, is highly susceptible to *F. oxysporum* f. sp. *cubense* race 4 (Warman, 2018).

The seeds for the six Chinese chive cultivars were purchased from Fusheng seed Co. in Henan province in China and were preserved in our laboratory. The sampling on the materials were conducted on our experimental field in Fujian Agriculture and Forestry University in Fujian province. The cultivar Duokang Fujiu 11 (DKF) shows strong resistance to stress and a strong special spicy odor. The Fujiubao (FJB) shows the strongest spicy odor among the six varieties but the lowest stress resistance. The Fujiuhuang 2 (FJH) shows a moderate spicy odor and stress resistance, its leaves are yellow (the rest were green). The Duokang Sijiqing (DKS) shows high resistance but almost no spicy odor. The Futaijiu 1 (FTJ) shows strong spicy odor but weak resistance and garlic sprout. The Zigenchun Zaohong (ZGC) shows a medium level of stress resistance and spicy odor but red roots (roots of the rest are white)**.**

### FOC fungal materials

The original fungal inocula of *F. oxysporum* f. sp. *cubense* race 4 (FOC) were kindly provided by Professor Zide Jiang of College of Agriculture at South China Agricultural University in Guangzhou [34]. The strain was maintained in Potato Dextrose Agar (PDA), and the inocula were cultured in PDA at 25 °C in darkness. PDA medium was prepared and sterilized by autoclaving at 121 °C for 20 min [5].

The FOC conidial suspension was prepared by incubating FOC in PDA broth on a shaker at 200 rpm and 28 °C for 5 days, followed by filtration with two layers of gauze to remove the mycelia. The conidial concentration was adjusted to 1 × 10^6^ conidia per milliliter [37].

### Inhibitory effect of leaf volatiles

To compare the inhibitory effect of the volatiles from the six Chinese chive cultivars, fresh leaves of six Chinese chive cultivars were cut into segments (about 1 cm long and 0.4 cm wide) or ground into powder in liquid nitrogen, respectively. Then the leaves were added in a four-room petri dish (90 mm in diameter) and inoculated with two 4 mm discs of FOC inocula. The mass of leaves was 0.6 g FW in the petri dish. Similar petri dish without Chinese chive leaves was used as controls. Six replicates were prepared for each treatment. Each six-petri dish containing the same sample were placed in the middle of a 4000 ml glass jar (the height and the diameters of top and bottom were 12.5, 22, and 19 cm, respectively).

The experiment was conducted in a climate-controlled room at 25 °C in darkness. Colony diameters of the inoculated FOC were measured with a Vernier caliper when the colony of the control had reached the peripheral wall.

### Inhibitory effect of aqueous leachates of the aerial parts

To examine the inhibitory effect of aqueous leachates of the six Chinese chive cultivars on FOC colony growth, the fresh aerial parts (50 g) were also ground into powder in liquid nitrogen and soaked in 100 mL distilled water at 25 °C for 20 min for extractions by an ultrasonic agitation, respectively (Zuo et al. 2016). Then, the aqueous solution was centrifuged at 10000 rpm for 5 min. After centrifugation, the supernatant was filtered first through normal filter paper (0.45 μm, Xingya Purification Factory, Shanghai, China) and then through a microfilter of pore size 0.22 μm (Whatman puradiscTM25AS polyethersulfone membrane, catalog NO.6794–2514, England). The final filtrate was our original leachates with a concentration of 0.5 g FW·mL^− 1^.

The filtered leachates (5 mL) of each cultivar and fungal culture medium (5 mL) were mixed and added in petri dishes (90 mm in diameter) and inoculated with a 4 mm discs of FOC. The concentration of final culture medium was 0.25 g FW·mL^− 1^. Similar petri dish without Chinese chive leachate but with the same volume of distilled water was used as controls. Six replicates were prepared for each treatment.

The experiment was conducted as described in the above methods part ‘Inhibitory effect of leaf volatiles’.

### Antifungal activities of DPT, DMT and MP

Based on our previous result, three compounds including DPT, DMT, and MP were used to test the antifungal activities against FOC colony growth. Four beakers (250 mL, 75 mm in diameter) filled with10 mL fungal culture medium of PDA were placed in a 4000 mL glass jar (the height and the diameters of top and bottom were 12.5, 22, and 19 cm, respectively), and inoculated with 4 mm disc of FOC. A piece of degreasing cotton filled with 100 μL the antifungal compounds (DPT, DMT, and MP) was placed in the middle of the jar. The final concentration of the three compounds was 25 μL·L^− 1^. Similar beakers in each glass jar without antifungal compound were used as controls. The glass jars were covered carefully with two layers of cellophane to stop the volatiles from escaping the [38]. Five replicates were prepared for each treatment. Thus, each treatment had 20 beakers placed in 5 glass jars.

The experiment was conducted in an incubator at 25 °C. Colony diameters of the inoculated FOC were measured with a ruler from the bottom by placing the glass jar on a high platform starting from 48 h post inoculation until 5 days when the colony of the control (no antifungal compound) breakers had grown close to the peripheral wall.

### Quantification of DPT, DMT and MP in the volatiles or aqueous leachates

To quantify DPT, DMT and MP in the volatiles released from Chinese chive cultivars, the volatiles were trapped according to the methods described by Zhang et al. [34]. The leaves of Chinese chives (150 g) were cut with 1 cm segments and added into a 500 mL flask. The volatiles were trapped through a glass tube (3 mm inner diam, 14 cm long) packed with 150 mg of Tenax TA (2.6–diphenylene oxide polymer resin) for 5 h. Trapped volatiles were eluted from each tube with 1 mL *n*-hexane and then were used for analysis by a gas chromatography system (GC) as described by Nandakumar et al. [38].

To quantify DPT, DMT and MP in the aqueous leachates of Chinese chive cultivars, the leaves (50 g) were ground into powder in liquid nitrogen and extracted with 100 mL distilled water at 25 °C for 20 min through an ultrasonic agitation, respectively. Then aqueous solution was centrifuged at 10000 rpm for 5 min. The supernatant (60 mL) was consecutively partitioned with dichloromethane (100 mL). The dichloromethane layer was separated with a separatory funnel, followed by evaporation under vacuum. The substance of dichloromethane phase was finally dissolved with dichloromethane to 5 mL. The final solution was filtrated by a micro filter with pore size 0.22 μm for and ready for GC analyses.

GC analyses were performed on an Agilent GC 7890B system (Agilent Technologies, Palo Alto, CA, USA) with a flame ionization detector (FID) and a capillary column Agilent 19,091 J-413 HP-5 (5% phenylmethyl siloxane, 30.0 m × 320 μm × 0.25 μm). The injection temperature was 150 °C, and the oven temperature was raised from 35 °C (2 min hold) to 250 °C at a rate of 10 °C·min^-1^. Two microliters of sample were injected, and nitrogen was used as the carrier gas at a flow rate of 1.0 mL·min^-1^.

### Banana intercropping with three Chinese chive cultivars in the pots

Based on the results of antifungal activities and quantity of the three active compounds, three cultivars (FJH, DKS, and DKF) of Chinese chive were selected to intercrop with banana in pots (the height and the diameters were 34 and 40 cm) filled with soils from the field of monoculture banana plantation for 6 years in Moxi Village, Tianbao Town of Nanjing County, Fujian Province (China, 24°29′14″ north latitude, 117°29′3″ longitude east). The monoculture banana in the same soil served as a control (CK). Thirty days after intercropping with Chinese chive under greenhouse conditions (25 ± 4 °C; 16/8 h light/dark photoperiod), each pot of all treatments (including CK treatment) was watered with 100 ml Fusarium conidial suspension (1 × 10^6^ spores·mL^− 1^) prepared as described above. The treatments and controls consisted of 10 plants per replicate.

Sixty days after fungal inoculation, plant height, stem circumference, photosynthesis parameters and disease indices were recorded. Net photosynthetic rate and stomatal conductance of the bananas were measured with Li-6400 (LI-COR, Lincoln, NE, USA). At the end of the experiment, each plant was harvested to assess the disease indices [39].

### Banana intercropping with three Chinese chive cultivars in the field

Seeds of three Chinese chive cultivars (FJH, DKS, and DKF) were sown in an experimental field in Moxi Village, Tianbao Town of Nanjing County, Fujian Province (China), in March of 2016 for 10 months planting. An experimental field that Fusarium wilt disease frequently-occurred in the village in January of 2017 was selected and divided into 20 plots each with a size of 2.5 × 25 m^2^ ([21]; Huang et al., 2016). Among the 20 plots, 5 plots were mono-cultured with banana plants (CK), and in the other 15 plots, banana plants were intercropped with the Chinese chive cultivars (FJH, DKS, and DKF) that had planted for 10 months at a spacing of 15 × 10 cm^2^. Five plots from another field nearby with no fusarium wilts disease occurred were also mono-cultured with banana plants (mock). Each plot of all treatments was planted with ten plants (at a spacing of 2.5 × 2.25 m^2^) of banana cultivar “Williams”. The planting site was prepared and managed using established cultivation practices, without the application of either herbicide or fungicides. Plants were fertilized twice a year by using NPK (N: P_2_O_5_: KCL = 10:3:2) with dose of 3 kg·plot ^− 1^. The incidence of Fusarium wilt disease of banana was examined every 30 days. When the banana ripened, fruit weight of each plant was weighed.

### Statistical analysis

Antifungal activities of three antifungal compounds, inhibitory effect of volatiles or aqueous leachates of the six Chinese chive cultivars on FOC were presented as inhibition rate (%) compared to control using the following formula: inhibition rate (%) = [(colony diameters of control - colony diameters of treatment) / colony diameters of control] × 100.

All data were subjected to analysis of variance using the Statistical Analysis System Program (SPSS 9.0). Each value was expressed as the mean ± the standard error (SE). Statistical significance was analyzed using Student’s *t*-test and one-way analysis of variance (ANOVA). The precision of the method was confirmed by least-significant difference (LSD, %). The values were considered significant when the *P* value was < 0.05.

## Results

### Inhibitory effect of volatiles from the six cultivars

**Fig. 1:**
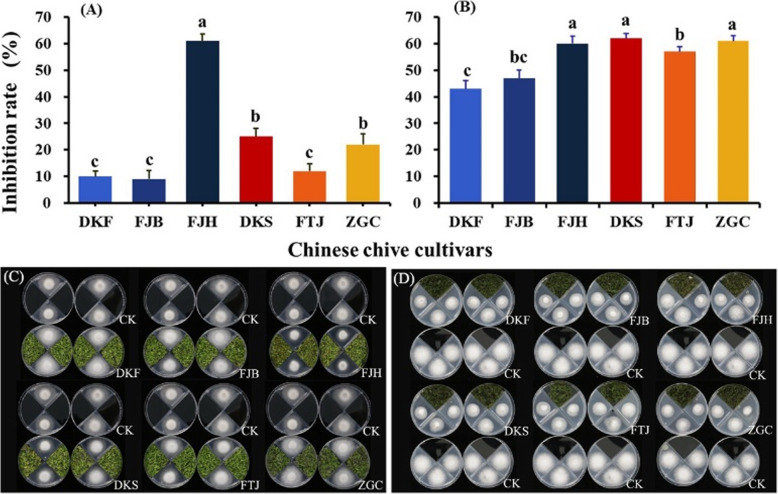
Inhibitory effects of volatiles released from sliced leaves (**a** and **c**) and the ground-leaves (**b** and **d**) of the six Chinese chive cultivars on colony growth of *Fusarium oxysporum* f. sp. cubense race 4 (FOC). The six cultivars are Duokang Fujiu 11 (DKF), Fujiubao (FJB), Fujiuhuang 2 (FJH), Duokang Sijiqing (DKS), Futaijiu 1 (FTJ), and Zigenchun Zaohong (ZZH). The concentration of volatiles was 0.6 g FW in the four-room petri dish (90 mm in diameter). Data are means ± SE (*n* = 12). Significant differences among treatments were indicated by different letters above the bars (*P* = 0.01 by Duncan’s multiple range test)

Fresh leaves of the six Chinese chive cultivars were treated in two ways: cut into 1 cm segments to acquire the naturally released volatiles, or ground into powder in liquid nitrogen to acquire maximally released volatiles. Bioassays showed that both the sliced leaves treatment and the ground-leaves treatment strongly inhibited the mycelial growth of FOC (Fig. 1).

In the sliced leaves treatment, the volatiles from all the six Chinese chive cultivars significantly inhibited mycelial growth of FOC (Fig. 1a). FJH cultivar with yellow leaves displayed the maximum inhibition (61%), which was much higher than the other five cultivars. The DKS and ZGC showed 25 and 22% inhibition, respectively. The other three cultivars showed marginal inhibitory effect (≤12%).

In the ground-leaves treatment, the inhibitory effect of volatiles of all cultivars was much higher than that in sliced leaves treatments (Fig. 1b). Three cultivars (FJH, ZGC and DKS) displayed 60–62% inhibition against FOC mycelia growth, which were significantly higher than the other three cultivars.

### Inhibitory effects of aqueous leachates of the six cultivars

**Fig. 2:**
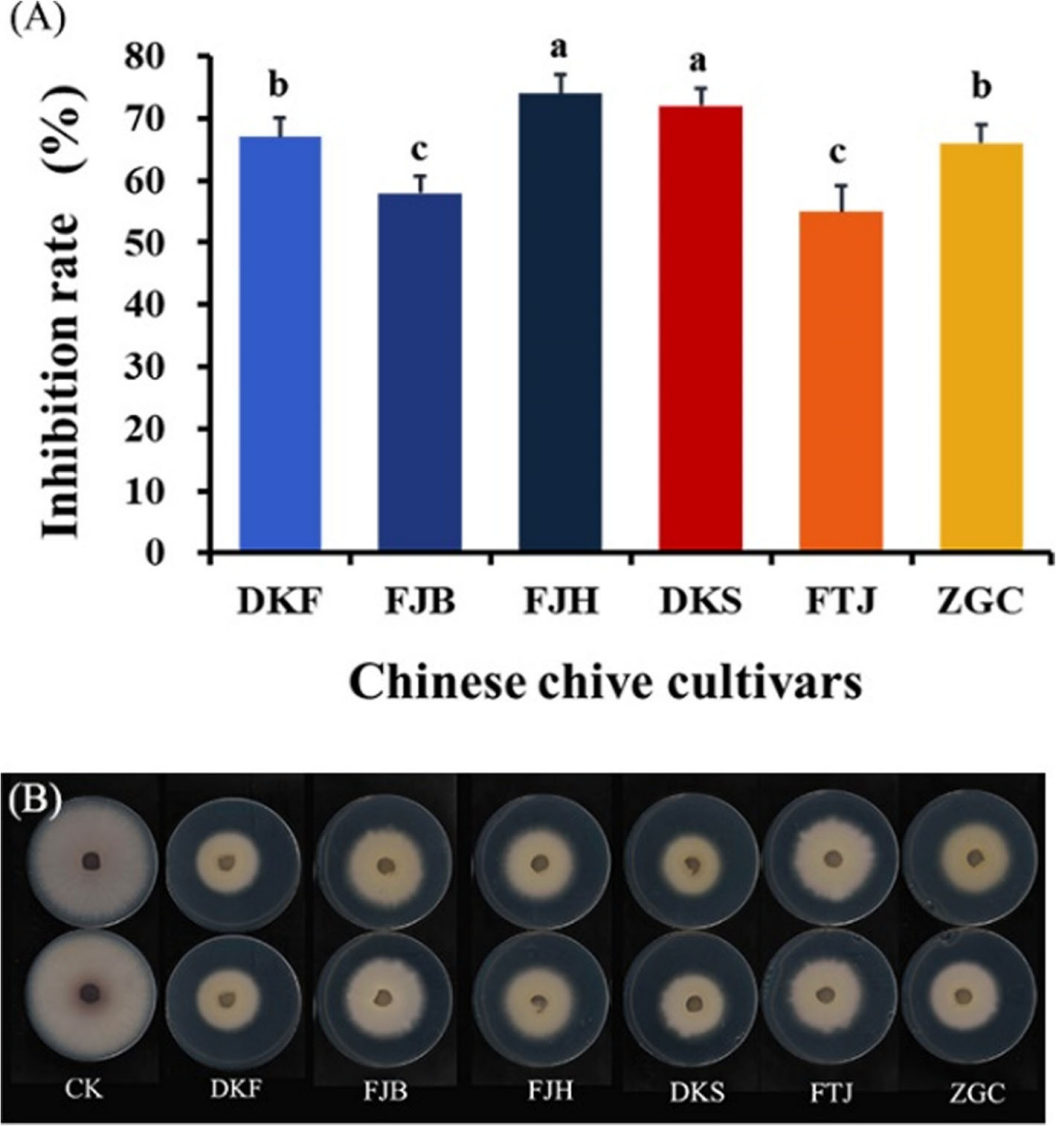
Statistical results (**a**) and petri dish exhibition (**b**) of inhibitory effects of aqueous leachates of aerial parts of six Chinese chive on colony growth of *Fusarium oxysporum* f. sp. cubense race 4 (FOC). The six cultivars were Futaijiu 1 (FTJ), Fujiubao (FJB), Fujiuhuang 2 (FJH), Duokang Sijiqing (DKS), Duokang Fujiu 11 (DKF) and Zigenchun Zaohong (ZGC). The concentration of aqueous leachates was 0.25 g FW·mL^− 1^. Data are means ± SE (*n* = 6). Significant differences among treatments were indicated by different letters above the bars (*P* = 0.01 by Duncan’s multiple range test)

The FOC colony growth in the presence of aqueous leachates of six Chinese chive cultivars is shown in Fig. 2. The aqueous leachates exhibited significant higher inhibitory effects (inhibition rate ranging from 55 to 72%) on FOC colony growth than the volatiles. The largest antifungal effect (72%) was obtained from the Chinese chive cultivar DKS, followed by FJH and DKF with inhibition of 68 and 67%, respectively (Fig. 2).

### Antifungal activity of DPT, DMT and MP

**Fig. 3:**
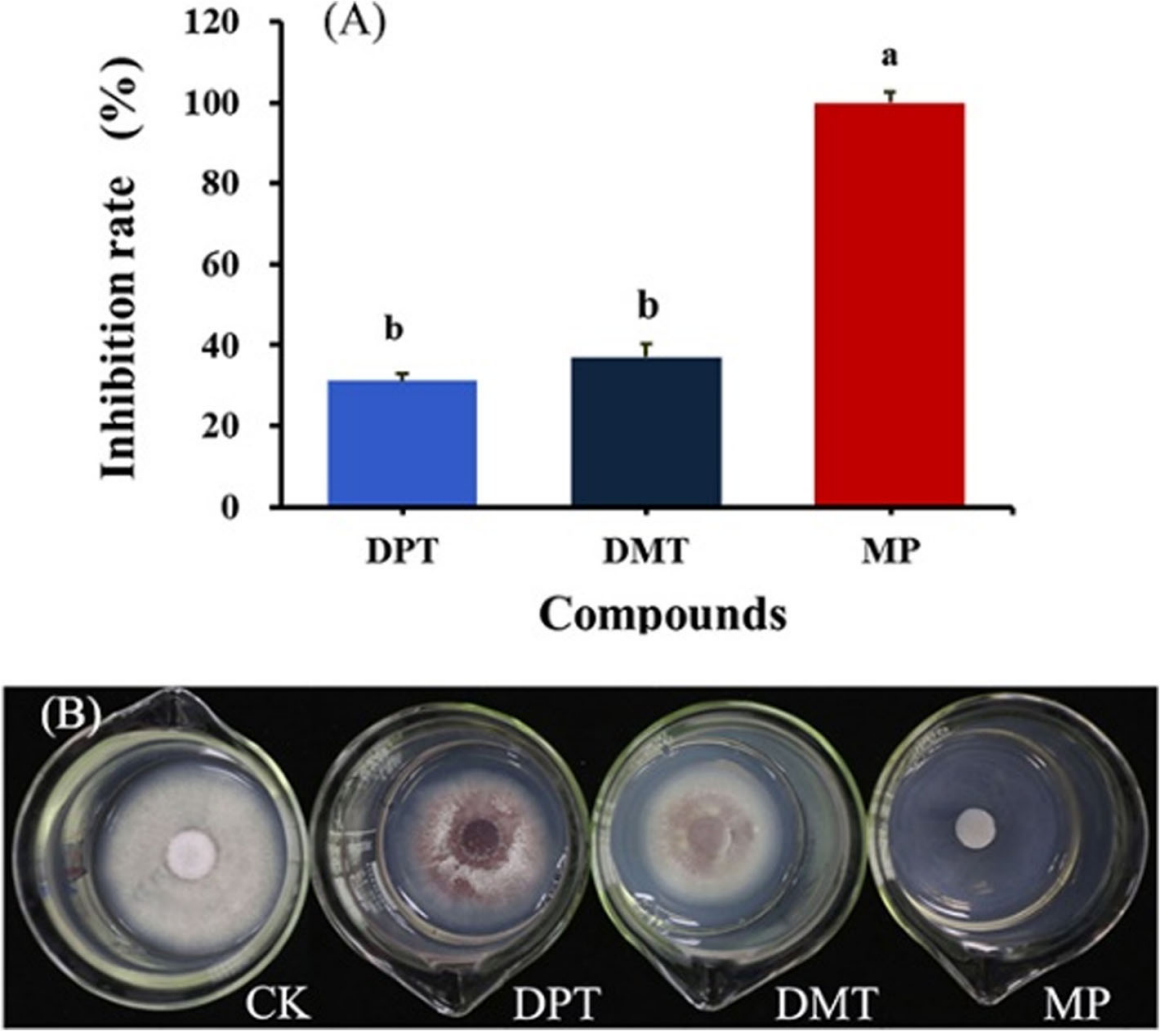
Statistical results (**a**) and experiment results exhibition (**b**) of antifungal activity of three compounds identified from Chinese chive against colony growth of *Fusarium oxysporum* f. sp. cubense race 4 (FOC)*.* The final concentration of the three compounds, 2-methyl-2-pentenal (MP), dimethyl trisulfide (DMT) and dipropyl trisulfide (DPT) was 25 μL·L^− 1^. Data are means ± SE (*n* = 20). Significant differences among treatments were indicated by different letters above the bars (*P* = 0.01 by Duncan’s multiple range test)

2-Methyl-2-pentenal (MP), dimethyl trisulfide (DMT) and dipropyl trisulfide (DPT) identified from Chinese chive significantly inhibited mycelial growth of FOC at a concentration of 25 μL·L^− 1^ (Fig. 3). MP almost completely inhibited the mycelial growth (99% inhibition), while DPT and DMT showed approximate 30% inhibition.

### Contents of MP, DMT and DPT in the six cultivars

**Fig. 4:**
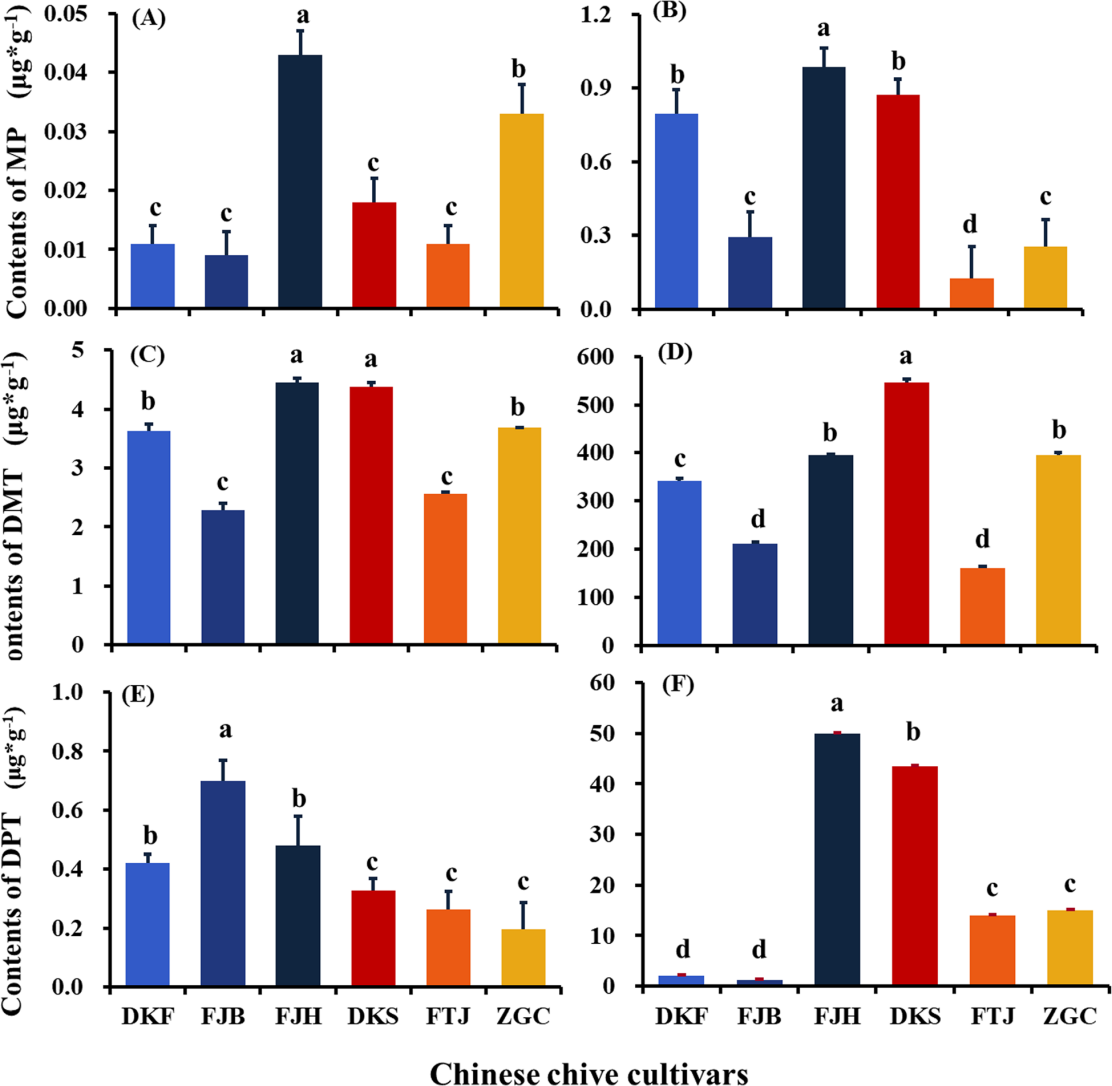
Contents of 2-methyl-2-pentenal (MP), dimethyl trisulfide (DMT), and dipropyl trisulfide (DPT) in the volatiles (**a**, **c**, **e**) and aqueous leachates (**b**, **d**, **f**) of the six Chinese chive cultivars. The six cultivars were Futaijiu 1 (FTJ), Fujiubao (FJB), Fujiuhuang 2 (FJH), Duokang Sijiqing (DKS), Duokang Fujiu 11 (DKF) and Zigenchun Zaohong (ZGC). Data are means ± SE (*n* = 3). Significant differences among treatments were indicated by different letters above the bars (*P* = 0.01 by Duncan’s multiple range test)

Since MP, DMT and DPT are important antifungal compounds in Chinese chive, and different chive species exhibited inconsistent inhibitory effect, we speculated that difference in antifungal activities among the six cultivars resulted from their difference in concentrations of DPT, DMT and MP. We, therefore, detected the contents of MP, DMT and DPT (Fig. 4) in the volatiles and aqueous leachates of the six Chinese chive cultivars.

The contents of MP, DMT and DPT significantly differed among the six Chinese chive cultivars. The highest concentrations of MP, the most active anti-FOC compound, was found in the cultivar FJH in both volatiles and leachates. The second most abundant MP was found in the volatiles of the cultivar ZGC and in the leachates of the cultivar DKS. DKS contained relative higher DPT, DMT and MP contents among all six chive species, while FJB and FTJ contained relative lower contents of these three antifungal substances (Fig. 4). For example, the content of DMT in the volatiles of FJH (4.454 g·g^− 1^), similar to that in DKS, was nearly two times as the content in FJB (2.282 μg·g^− 1^). This might explain that FJH and DKS had better performances in antifungal assays. Although DKF released less DMT and DPT than ZGC in aqueous leachates (Fig. 4), its anti-FOC activity was high, which might result from the higher contents of MP in aqueous leachates of DKF (0.794 g·g^− 1^) that was significantly higher than ZGC (0.253 g·g^− 1^) (Fig. 4).

### Intercropping improved banana growth and photosynthesis in the pots

Based on antifungal activity against FOC, three Chinese chive cultivars DKF, FJH and DKS with higher contents of DPT, DMT and MP, were selected for intercropping with banana in pot experiment to examine their differential effects on Panama disease.

**Fig. 5:**
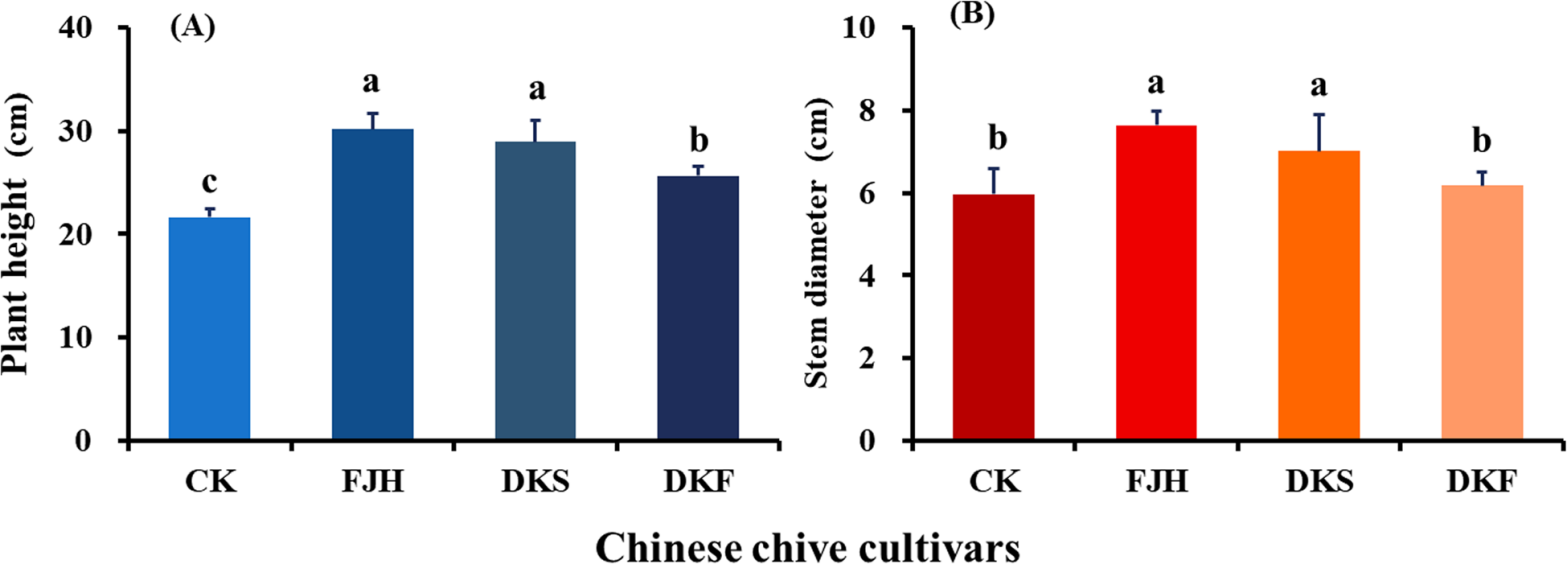
Vegetative growth of banana plants intercropping with three Chinese chive cultivars in pot experiment. **a** Plant height, (**b**) stem diameter. The three cultivars used for intercropping with banana were Fujiuhuang 2 (FJH), Duokang Sijiqing (DKS) and Duokang Fujiu 11 (DKF). The monoculture banana in the same soil served as a control (CK). Data are means ± SE (*n* = 10). Significant differences among treatments were indicated by different letters above the bars (*P* = 0.01 by Duncan’s multiple range test)

Ninety days after intercropping with Chinese chive, plant heights of banana plants were significantly improved by all three cultivars relative to monoculture control. Stem diameters were significantly improved by intercropping with FJH and DKS (Fig. 5).

**Fig. 6:**
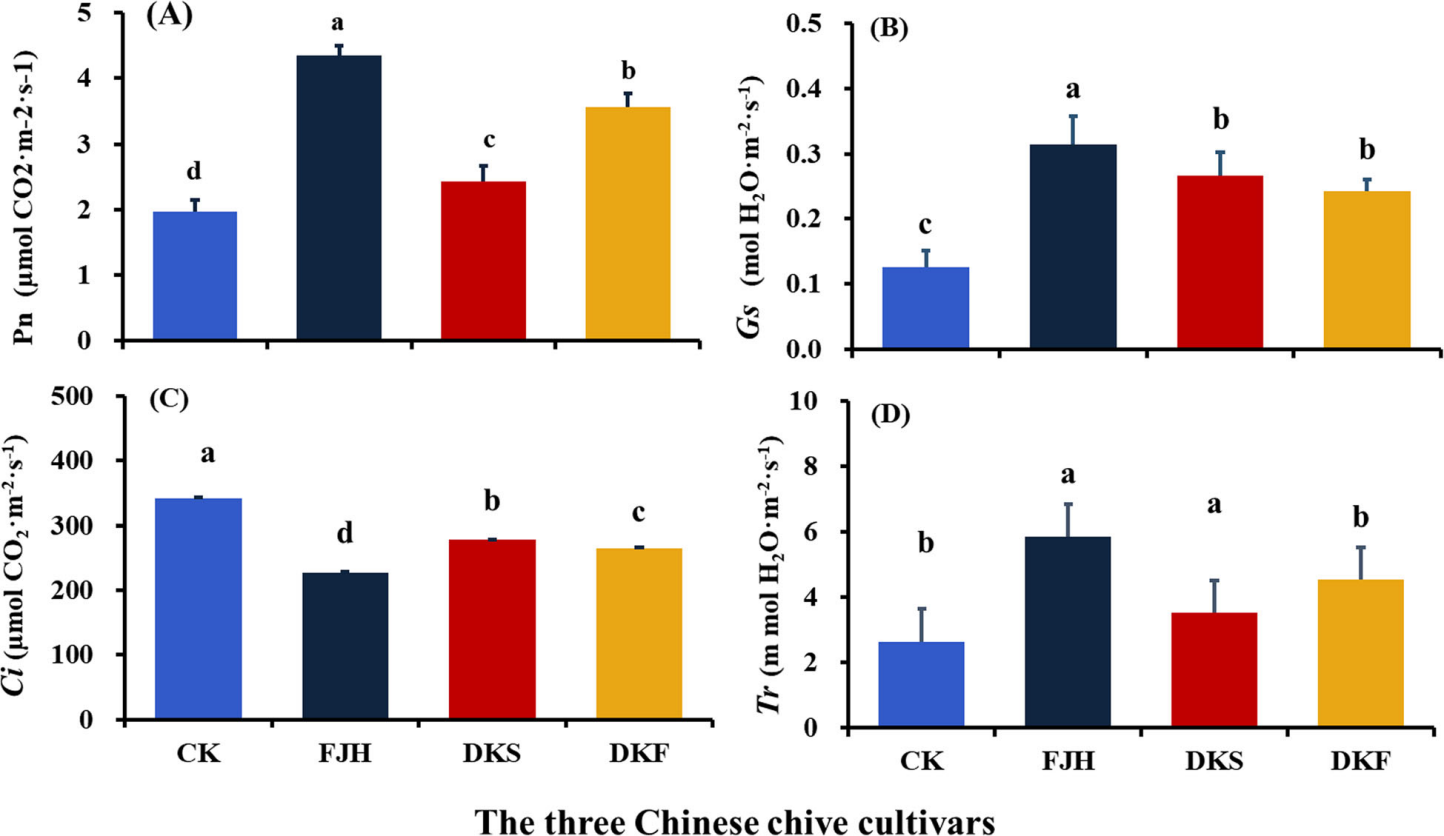
Net photosynthetic rate (*Pn*, **a**), stomatal conductance (*Gs*, **b**), intercellular CO2 concentration (*Ci*, **c**), and transpiration rate (*Tr*, **d**) of bananas plants intercropping with three Chinese chives cultivars in pot experiment. The three cultivars used for intercropping with banana were Fujiuhuang 2 (FJH), Duokang Sijiqing (DKS) and Duokang Fujiu 11 (DKF). The monoculture banana in the same soil served as a control (CK). The photosynthetic parameters were measured 60 days after FOC inoculation. Data are means ± SE (*n* = 10). Significant differences among treatments were indicated by different letters above the bars (*P* = 0.01 by Duncan’s multiple range test)

As shown in Fig. 6, the photosynthetic characteristics of banana functional leaves including net photosynthetic rate (*Pn*), stomatal conductance (*Gs*), and transpiration rate (*Tr*) in intercropping system were significantly higher than those in the monoculture system. Obviously, intercropping with all three cultivars significantly improved net photosynthetic rate and stomatal conductance of banana plants, but reduced intercellular CO_2_ concentration relative to monoculture banana plants (Fig. 6). Intercropping with FJH and DKS also significantly enhanced transpiration rate. Intercropping with DKF did not have obvious effect on transpiration rate. Furthermore, the cultivar FJH showed the strongest effects on the four photosynthetic parameters in the intercropping system.

### Intercropping reduced the Fusarium wilt incidence of banana in the pots

**Fig. 7:**
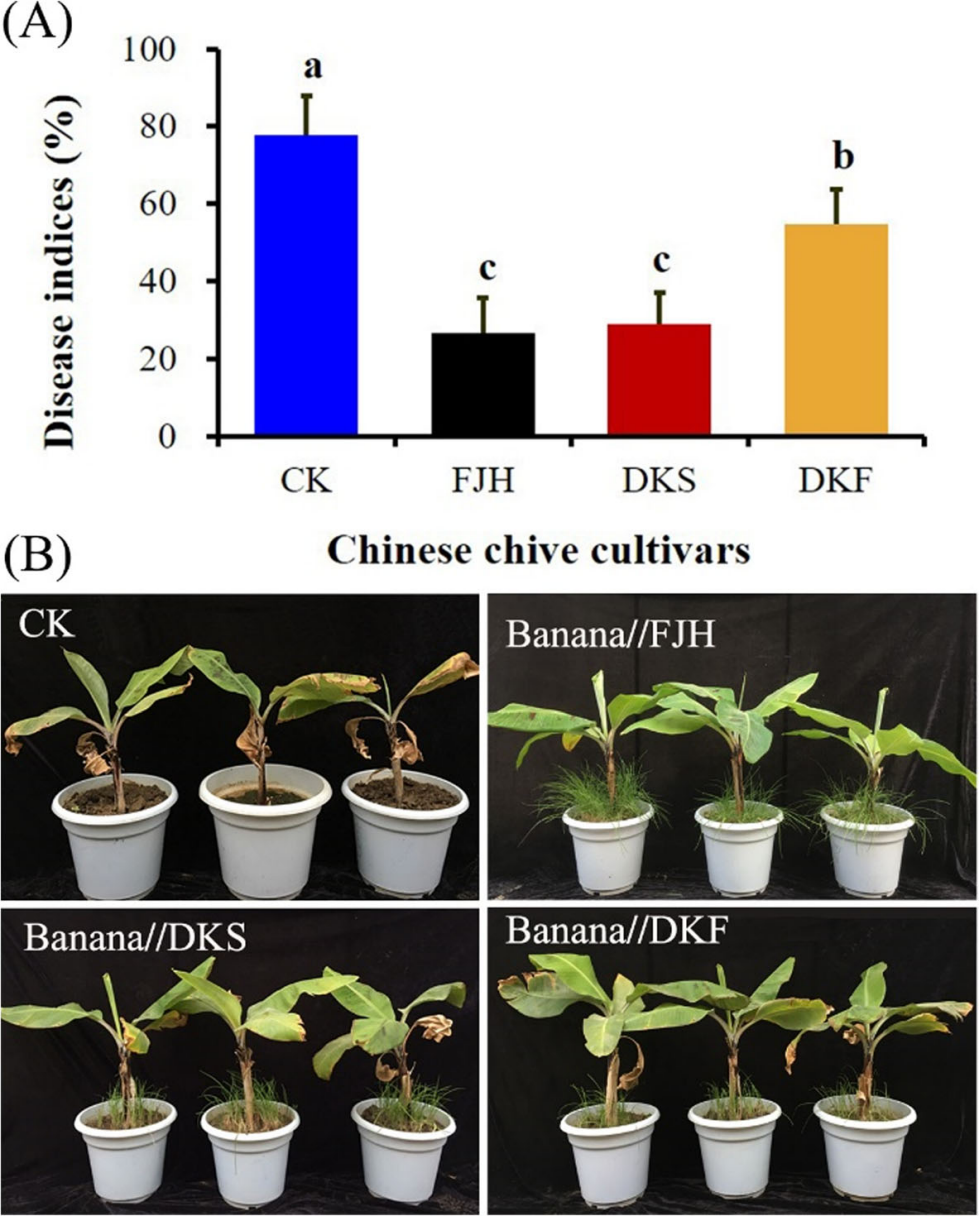
Fusarium wilt disease incidence (**a**) and growth performance (**b**) of banana plants 60 days after inoculation with of *Fusarium oxysporum* f. sp. cubense race 4 (FOC) in monoculture and intercropping with three Chinese chive cultivars in the pots. The three cultivars used for intercropping with banana were Fujiuhuang 2 (FJH), Duokang Sijiqing (DKS) and Duokang Fujiu 11 (DKF). The monoculture banana in the same soil served as a control (CK). Data are means ± SE (*n* = 10). Significant differences among treatments were indicated by different letters above the bars (*P* = 0.01 by Duncan’s multiple range test)

The occurrence of Panama disease of banana was significantly reduced after intercropping with all three cultivars of Chinese chive relative to monoculture banana plants (Fig. 7a). Intercropping with FJH, DKS and DKF reduced the disease indices by 65.7, 62.8 and 29.8%, respectively. The lowest disease indices (Fig. 7a) and very few yellow leaf symptoms were found in the banana plants intercropping with FJH.

### Intercropping reduced the Fusarium wilt disease incidence but increased banana yield in the field

**Fig. 8:**
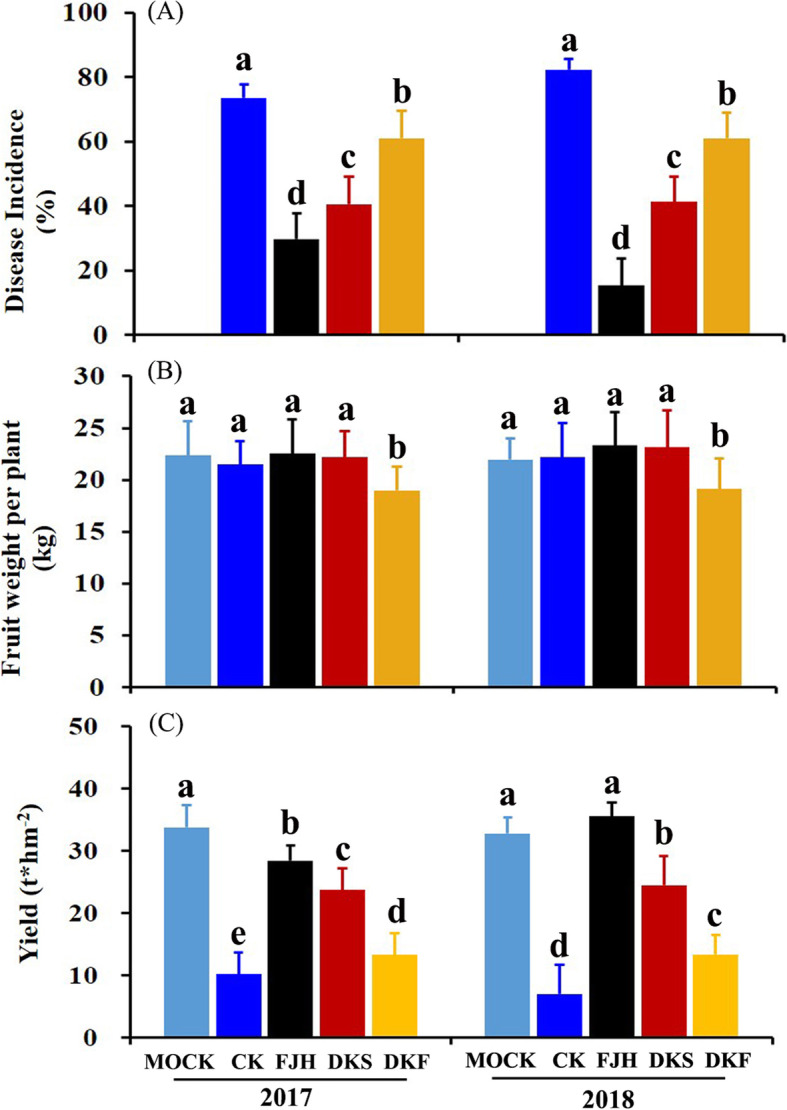
Fusarium wilt disease incidence (**a**), fruit weight (**b**) and yield (**c**) of banana plants after FOC inoculation in monoculture and intercropping with three Chinese chive cultivars in the field. The three cultivars used for intercropping with banana were Fujiuhuang 2 (FJH), Duokang Sijiqing (DKS) and Duokang Fujiu 11 (DKF). The monoculture banana in the same soil served as a control (CK). Another field nearby with no fusarium wilts disease occurred were also mono-cultured with banana plants (mock). Data are means ± SE (*n* = 5). Significant differences among treatments were indicated by different letters above the bars (*P* = 0.01 by Duncan’s multiple range test)

Field experiments were conducted in 2017 and 2018 to evaluate the effects of intercropping with the three selected Chinese chive cultivars on banana resistance to Fusarium wilt, as well as fruit weight per plant and yield (Fig. 8). Intercropping with all three Chinese chives cultivars significantly reduced the disease incidence but increased banana yield in comparison with the monoculture control (Fig. 8a & c). The lowest disease incidence and highest yield were found in banana plants intercropping with the cultivar Fujiuhuang 2 (FJH), followed by the cultivar DKS in both 2017 and 2018. Intercropping with FJH and DKS increased the yield by 5.04- and 2.78-fold, respectively (Fig. 8c). Intercropping with DKF displayed the weakest effect on both banana disease incidence and yield. However, the intercropping with FJH and DKS showed no obvious effects on fresh weight of each plant (Fig. 8b). Only DKF displayed a bit negative effect on fresh weight of each plant.

## Discussion

Panama disease severely affects banana production worldwide. Appropriate agricultural practices show potentials to successfully minimize the detrimental effect of the disease [9, 10, 40, 41]. Rotation and intercropping with Chinese chive have been demonstrated to be effective approaches to control the disease at both greenhouse and field levels [31, 34, 35]. This study showed that the cultivar selection is a key factor for successful control of the disease. Although all cultivars displayed obvious inhibition to the disease in both greenhouse and field conditions (Figs. 7 & 8), they showed significant difference in their ability to reduce the disease incidence. Two cultivars FJH and DKS exhibited much stronger inhibition against Panama disease. In particular, the cultivar FJH showed the highest inhibition to the disease and best improvement for the banana yield in both pot and field experiments (Figs. 7 & 8). Interestingly, in laboratory both volatiles and aqueous leachates of FJH showed the strongest inhibitory effect on the pathogen FOC among the six cultivars tested (Figs. 1 & 2). Meanwhile, the cultivar FJH produced the highest amount of 2-methyl-2-pentenal in both volatiles and aqueous leachates (Fig. 4a & b), which showed the strongest antifungal activity against FOC (Fig. 3 [34];). All results from laboratory, greenhouse and field experiments suggested that the cultivar FJH is the best cultivar of Chinese chive for intercropping with banana to control Panama disease. The second better cultivar is DKS, which produced the second abundant 2-methyl-2-pentenal in the aqueous leachate (Fig. 4b), and showed the second strongest antifungal activity against FOC in the leachate (Fig. 2). Schulz et al. [42] revealed the influence of the genetic background of the parent cultivars on the sulfur volatile composition in the *Allium* hybrid. Therefore, selection of appropriate cultivars of Chinese chive with strong anti-FOC activity for intercropping is a key for successful control of Panama disease. More cultivars should be screened to find suitable ones for intercropping with banana.

Volatile compounds released from host plants play an important role in plant defense against microbial pathogens [43]. *Allium* species are well-known to produce many volatile organosulfur compounds, which contribute to the unique smell and taste of these plant species [38, 44]. These compounds exhibit broad-spectrum biological activities towards microorganisms, plants and animals [44, 45]. Although this study showed that aqueous leachates displayed much higher anti-FOC activity, the active compounds in the aqueous leachates were volatiles and low polarity [34, 46], which is consistent with the result that the aqueous leachates had significantly more active compounds than volatiles (Fig. 4). The results indicated that anti-FOC compounds such as 2-methyl-2-pentenal and dimethyl trisulfide are easily released by Chinese chive via leaching and root exudation. Lian et al. [46] demonstrated that the aqueous leachates of Chinese chive roots were more inhibitory to FOC than those of stems and leaves. Zhang et al. [34] revealed that volatiles excluded by roots of Chinese chive inhibited spore germination of FOC. It is likely that in the field most active compounds of Chinese chive are released into the rhizospheres and then these volatiles spread into the soils to exhibit anti-FOC activity.

Volatile sulfur compounds have been demonstrated to be inhibitory to *Fusarium* and other pathogenic fungi [47]. Non-sulfur-containing compound 2-methyl-2-pentenal, a degradation product of thiopropanal S-oxide [48], displayed the strongest anti-FOC activity (Fig. 4). The chemical was also found in the onion (*Allium cepa* L.) essential oil and displayed antifungal activity to *Fusarium* [47].

Anti-FOC activity and contents of 2-methyl-2-pentenal in the volatiles and aqueous leachates may serve as two desired parameters for preliminary selection of appropriate cultivars of Chinese chive for intercropping with banana.

## Conclusion

Panama disease (Fusarium wilt disease) caused by *F. oxysporum* f. sp. *cubense* race 4 (FOC) severely threatens banana (Musa spp.) production worldwide. Intercropping of banana with Chinese chive can effectively reduce Panama disease.

Our study demonstrates that banana intercropping with Chinese chive can effectively reduce Panama disease and selection of appropriate cultivars of Chinese chive for intercropping is key for the success. The six cultivars showed significant differences in antifungal activity against FOC mycelia growth in both leaf volatiles and aqueous leachates. Pot and field experiments showed that intercropping with three selected Chinese chive cultivars significantly improved banana vegetative growth, increased photosynthetic characteristics and yield but decreased disease incidence of Panama disease. Among the six cultivars used FJH and DKS are the most suitable cultivars for intercropping.

## Data Availability

All data generated or analysed during this study are included in this published article.

## Abbreviations

CK: Control
DKF: Duokang fujiu 11
DKS: Duokang sijiqing
DMT: Dimethyl trisulfide
DPT: Dipropyl trisulfide
FJB: Fujiubao
FJH: Fujiuhuang 2
FOC: *Fusarium oxysporum* f. sp. *cubense* race 4
FTJ: Futaijiu 1
Gs: Stomatal conductance
MP: 2-Methyl-2-pentenal
PDA: Potato dextrose agar
Pn: Net photosynthetic rate
SE: Standard error
Tr: Transpiration rate
ZGC: Zigenchun zaohong
